# HPV-driven transcriptome and splicing rewiring under SRPK1 inhibition in cervical cancer

**DOI:** 10.3389/fonc.2025.1712170

**Published:** 2026-01-02

**Authors:** Afra Basera, Mohammed Alaouna, Janie Duvenhage, David O. Bates, Zodwa Dlamini, Rahaba Marima

**Affiliations:** 1 SAMRC Precision Oncology Research Unit (PORU), DSTI/NRF SARChI Chair in Precision Oncology and Cancer Prevention, Pan African Cancer Research Institute (PACRI), University of Pretoria, Hatfield, Pretoria, South Africa; 2 Department of Medical Oncology, Faculty of Health Sciences, Steve Biko Academic Hospital, University of Pretoria, Pretoria, South Africa; 3 Nuclear Medicine Research Infrastructure NPC, Pretoria, South Africa; 4 Radiochemistry, the South African Nuclear Energy Corporation (Necsa) SOC Ltd, Pelindaba, South Africa; 5 Center for Cancer Sciences, BioDiscovery Institute, University Park, Nottingham, United Kingdom; 6 Wolfson Wohl Cancer Research Centre, School of Cancer Sciences, University of Glasgow, Garscube Estate, Glasgow, United Kingdom

**Keywords:** SRPK1, transcriptome remodeling, exon skipping (SE), intron retention (RI), alternative 3′ splice site (A3SS), alternative 5′ splice site (A5SS), mutually exclusive exons (MXE), SPHINX31

## Abstract

**Background:**

Serine/arginine protein kinase 1 phosphorylates serine-arginine-rich (SR) proteins to regulate splice-site selection during alternative splicing. While its role in general RNA regulation is established, its contribution to the HPV-dependent transcriptome and splicing stratification in cervical cancer remains unclear. Therefore, we sought to determine how SRPK1 inhibition differentially remodels gene expression and alternative splicing in HPV^+^ versus HPV^-^ cervical cancer cells.

**Methods:**

HPV16^+^ SiHa and HPV^-^ C33A cervical cancer cells were treated with the SRPK1 inhibitor, SPHINX31. RNA profiling was performed, and differentially expressed genes were defined as |log_2_FC| ≥ 1.5. AS events were classified by SUPPA as exon skipping (SE), intron retention (RI), mutually exclusive exons (MXE), alternative 3′ splice site (A3SS), and alternative 5′ splice site (A5SS). Pathway enrichment was assessed using Gene Ontology/KEGG, STRING protein-protein interaction (PPI) networks, and Molecular Complex Detection (MCODE) was used to identify protein hubs. To determine computational prediction of docking, SPHINX31 was docked into SRPK1 (PDB 5MY8) using SP/XP docking and MM-GBSA rescoring.

**Results:**

SRPK1 inhibition was associated with distinct responses that were HPV-related. In C33A cells, upregulated genes were enriched for translation, RNA processing, and glycosylation, with KEGG highlighting ribosome and metabolic modules. Ribosomal hubs dominated the PPI/MCODE, suggesting possible translational and metabolic adjustments. In contrast, SiHa cells exhibited transcriptomic changes consistent with reduced expression of genes linked to Hippo, Wnt, PI3K-AKT, ERK1/2 signaling, migration, angiogenesis, and growth factor cytokine networks. Targets of YAP/TAZ (e.g., *CCND1, BIRC5, SNAI2, SERPINE1*) and their regulators (*RASSF1, CSNK1E*) were suppressed. At the splicing level, SiHa cells displayed fewer total AS events but with larger effect sizes, particularly in A3SS/A5SS. C33A cells showed abundant SE (59,234 events; small median ΔPSI) and RI (1,770 events, often binary), including complete shifts in *HLA-DRB1/PLIN2* (+1.00) and KLF4 (-1.00). Notable A5SS switches included *LEF1* (+1.00) and *CDK6* (-1.00) in C33A, and *DLX1/MRPL14/THAP5* (-1.00) in SiHa. Docking computationally predicted the low-energy poses of SPHINX31 in the SRPK1 ATP pocket. While not definitive, this evidence may potentially support the transcriptomic and splicing findings.

**Conclusion:**

SRPK1 inhibition may remodel the cervical cancer transcriptome in an HPV-linked manner, with SiHa cells exhibiting changes consistent with suppression of oncogenic signaling, whereas C33A cells adapt through translational and metabolic reprogramming.

## 1 Introduction

Cervical Cancer (CCa) ranks as the second most prevalent malignancy among women globally and a major cause of mortality in low- and middle-income countries (1, 2). Persistent infection by the high-risk human papillomavirus (HPV), specifically HPV16/18, is the primary cause of CCa, mainly through the viral E6 and E7 proteins that alter tumour suppressive pathways (3–8). Despite screening and vaccine programs reducing the incidence of CCa, novel treatment strategies are required for advanced disease states and recurrence (9).

An emerging hallmark of HPV-related cancer is aberrant RNA splicing (10). Alternative splicing (AS) is a distinct cellular mechanism in eukaryotes that produces several transcripts from a single gene, increasing proteomic diversity. Introns are removed, and exons are connected to produce proteins with various functions, including migration, maturation, differentiation, and apoptosis (11). The spliceosome promotes this process (12). Dysregulated pathways, coupled with HPV infection, are essential for CCa progression and development. CCa shows widespread AS dysregulation alongside gene expression (13–15). Splicing factors promote CCa by facilitating HPV RNA maturation (supporting viral replication/oncoprotein production) and producing tumor-promoting host isoforms (16, 17). Serine-arginine (SR) proteins are highly conserved splicing factors that have one or two RNA recognition motifs (RRMs) at the N-terminal and an SR dipeptide highly repetitive domain (RS domain) at the C-terminal (18). SR proteins are crucial in spliceosome maturation, splice site choice, AS, and RNA metabolism, such as mRNA export, translation, localization, and nonsense-mediated RNA decay (19–21). The location and activities of SR proteins are primarily presided over by reversible phosphorylation within the RS domain (22), and the sequential phosphorylation modification process is mainly carried out by two protein kinase families: serine-arginine protein kinases (SRPKs) (23, 24) and cdc2-like kinases (CLKs) (25).

SRPKs phosphorylate SR proteins in the cytoplasm, leading to their movement to the nucleus and assembly in interchromatin speckles (26). Serine/arginine protein kinase 1 (SRPK1) regulates AS by phosphorylating several SRSFs rich in SR domains and regulates the movement of SRSFs within the nucleus during mitosis, rearranging nuclear speckles (24, 27, 28). Alterations in splicing modulators and suppression of their activity may lead to abnormal AS, which drives several malignancies (29–35). Current studies indicate that HPV16 infection elevates SRPK1 expression and facilitates splicing factor phosphorylation, mainly through the viral E2 oncoprotein, implying virus-mediated splicing reprogramming (36, 37). In CCa, SRPK1 upregulation encourages cell growth, migration, and invasion and is linked to progression and unfavorable patient outcomes in cervical squamous cell carcinoma (35).

Pharmacological agents targeting several spliceosome machinery constituents or modulators are currently gaining attention as potential anti-cancer medications. One such inhibitor is SPHINX31, which has been modified from SRPIN340, which targets SRPK1 and has demonstrated efficacy in inhibiting SRPK1 in various cancer models (38, 39). However, the transcriptome-wide impact of SRPK1 inhibition in HPV-associated CCa remains unexplored. This study used RNA sequencing to profile gene expression and AS changes in HPV-positive and HPV-negative CCa cell lines following treatment with SPHINX31. By identifying differential splice variants and disrupted pathways, we aimed to uncover SRPK1-driven splicing events with potential relevance to tumor biology and therapeutic targeting.

## 2 Materials and methods

### 2.1 Cell culture

C33A (HPV- negative epithelial cell line) and SiHa (HPV16^+^ epithelial cell line) cell lines were used in this study. The cells were grown in flasks with Dulbecco’s Modified Eagle’s Medium (DMEM) with 10% Fetal Bovine Serum (FBS) (Thermo Fisher Scientific, Waltham, MA, USA) and 1% Penicillin-Streptomycin antibiotic (Thermo Fisher Scientific, Massachusetts, USA) and incubated at 37°C in a humidified incubator.

### 2.2 Drug treatment

Cells were seeded into a flask and allowed to reach 70-80% confluency and treated with 3μM SPHINX31 (MedChemExpress, New Jersey, USA) or vehicle control (0.1% dimethyl sulfoxide, DMSO) for 48 hr. The exposure was selected based on prior evidence of biological efficacy 3μM (40–42).

### 2.3 Next generation sequencing

To determine transcriptomic changes, RNA was extracted using the Qiagen RNeasy Mini Kit (Qiagen, Hilden, Germany) from 4 samples (2 SiHa samples and 2 C33A samples) according to the manufacturer’s instructions and quality controlled using Qubit and Agilent TapeStation, with samples achieving an RNA integrity number (RIN) of > 7.0. Ribosomal RNA depletion was performed using the MGIEasy rRNA Depletion Kit (MGI Tech, Shenzhen, China). This approach was selected to maximize coverage of coding and non-coding transcripts for transcriptome-wide expression and splicing analysis, although the study primarily analyzed protein-coding genes. Sequencing libraries were constructed using the MGIEasy RNA Library Prep kit (MGI Tech, Shenzhen, China) according to the manufacturer’s instructions. Following second-strand synthesis, cDNA was cleaned using MGIEasy DNA Clean Beads and A-tailed. Adapters were then added to the A-tailed samples using the MGIEasy DNA Adapters-96 (Plate) kit. Subsequently, the samples were washed using DNA Clean Beads, and PCR amplification was performed. The quality and quantity of the PCR products were evaluated using Qubit and Agilent TapeStation. Following amplification, the PCR product was denatured for multiplexing and subsequently circularized using the MGIEasy Circularization Module. Paired-end sequencing was carried out using the MGI DNBSEQ−2000 system with a target depth of ~30 million reads.

### 2.4 Read alignment and differential gene expression analysis.

To quantify gene expression and identify DEGs, Raw FASTQ files were quality-checked using FastQC within Galaxy Europe (v22.05) (galaxyproject.eu). Adapter trimming and low-quality read removal were performed using Trimmomatic (v0.39). High-quality reads were aligned to the human reference genome (GRCh38/hg38, GENCODE v38 annotation) using HISAT2 v2.2.1. The total number of reads that aligned to the exons of each gene in the human genome, as defined by GENCODE version 38, was obtained using HTSeq v0.13.5. Detailed command lines, parameter settings, and version information are provided in the supplementary methods to ensure full reproducibility (Supplementary Methods 4). Given that RNA-seq was performed with a single independent biological sample per condition (n = 1) and per cell line (DMSO-treated and drug-treated for each cell line), statistical significance or p-values were not computed. Differential expression was calculated using log 2fold-change relative to the control, with a threshold set at |log_2_FC|≥1.5. Since SiHa and C33A differ in genetic background (including p53 status) and HPV genotype, each cell line was treated and analyzed independently with its own matched control. The comparative interpretation, therefore reflects HPV-contextual rather than strictly isogenic differences.

### 2.5 Function enrichment analysis

Gene ontology term and KEGG pathway enrichment analyses were performed using the Database for Annotation, Visualization, and Integrated Discovery (DAVID). Because the dataset consisted of single biological replicates, per-gene statistical testing was not applicable. DEGs were defined solely by an absolute log_2_ fold change (|log_2_FC| ≥ 1.5). Pathway enrichment was performed using DAVID which applies a Benjamini-Hochberg FDR correction to enrichment p-values derived from hypergeometric tests of overlap between the DEG list and functional gene sets. Consequently, reported FDR values correspond to pathway-level significance rather than individual gene-level statistics. Differentially expressed genes (DEGs) identified in CCa were submitted without a custom background list, allowing DAVID to default to the full human genome as a reference.

### 2.6 Protein-protein interactions network and hub analysis of DEGs

The Search Tool for the Retrieval of Interacting Genes/Proteins (STRING) database (Version: 12.0) (https://string-db.org/) software was used to search for proteins and determine the PPIs between the proteins encoded by the dysregulated genes. (43) The DEGs for the cell lines were uploaded into the database to obtain the PPIs. The PPI network comprises nodes and edges that represent proteins and interactions. The thickness of the edge indicates strong interactions between proteins. A medium STRING interaction threshold of > 0.40 was considered significant, and DEGs without interactions were removed. Following functional enrichment analysis of the DEGs to identify significant biological pathways, a PPI network was constructed to explore the potential interaction patterns among the dysregulated genes. The PPI network was visualized using Cytoscape (https://cytoscape.org/; version 3.10.3). The Molecular Complex Detection (MCODE) plugin in Cytoscape was used to identify significant modules in the PPI network. Modules were detected based on node density and connectivity using the default parameters (degree cutoff = 2, node score cutoff = 0.2, K-core = 2, and max depth = 100). The resulting clusters were used to explore functionally coherent gene groups within the PPI network and to reduce noise.

### 2.7 Alternative splicing analysis

RNA-seq reads were first aligned to the human reference genome (GRCh38/hg38) using HISAT2 v2.2.1, with default parameters. The resulting BAM alignment files were sorted and indexed using SAMtools v1.10 to ensure compatibility with downstream quantification. Transcript abundance was estimated using StringTie v2.2.1 in transcript-guided mode with the GENCODE v38 annotation (GTF). StringTie v2.2.1 generated transcript-level TPM expression matrices that served as inputs for the splicing analysis. Alternative splicing events were analyzed using SUPPA2 (version 2.3; (44)). Event definitions (IOE files) and transcript expression (TPM) values were generated from GENCODE v38 (GRCh38) annotations to identify five major splicing types: skipped exons (SE), retained introns (RI), mutually exclusive exons (MXE), and alternative 3′ and 5′ splice sites (A3SS, A5SS).

PSI (Percent Spliced In) values were calculated for each event using SUPPA2’s psiPerEvent function, which normalizes inclusion and exclusion isoform expression by total event expression. Events were retained only when the denominator (sum of inclusion and exclusion TPMs) ≥ 1.0 in both conditions, and those with undefined or zero denominators were automatically excluded. Visualization of the top 10 events per category was performed in Python 3.10 using matplotlib and seaborn. Event-level schematics were generated to illustrate exon inclusion and exclusion for each major splicing type. For comparative analysis, ΔPSI was computed as PSITreated-PSI Untreated. Events were summarized at the event level, and those with the largest absolute ΔPSI values were prioritized for further analysis. Visualization was performed using volcano-like scatter plots, in which ΔPSI was plotted against log10 (mean denominator + 1) to emphasize both effect size and event support. The top 10 events per plot were annotated to avoid label overlaps. Visualization was performed using volcano-like scatter plots, in which ΔPSI was plotted against log10 (mean denominator + 1) to emphasize both effect size and event support. The top 10 events per plot were annotated to avoid label overlaps. Given the use of single biological replicates (n = 1) and limited RNA, wet-lab validation (RT-PCR) was not performed at this stage.

### 2.8 Statistical analysis

Computational analysis was performed using R (v4.3.0) and Python. The results are reported as |log_2_ fold change| instead of statistical significance.

## 3 Results

### 3.1 SRPK1 inhibition alters gene expression in HPV-negative and HPV-positive CCa cells

**Figure 1:**
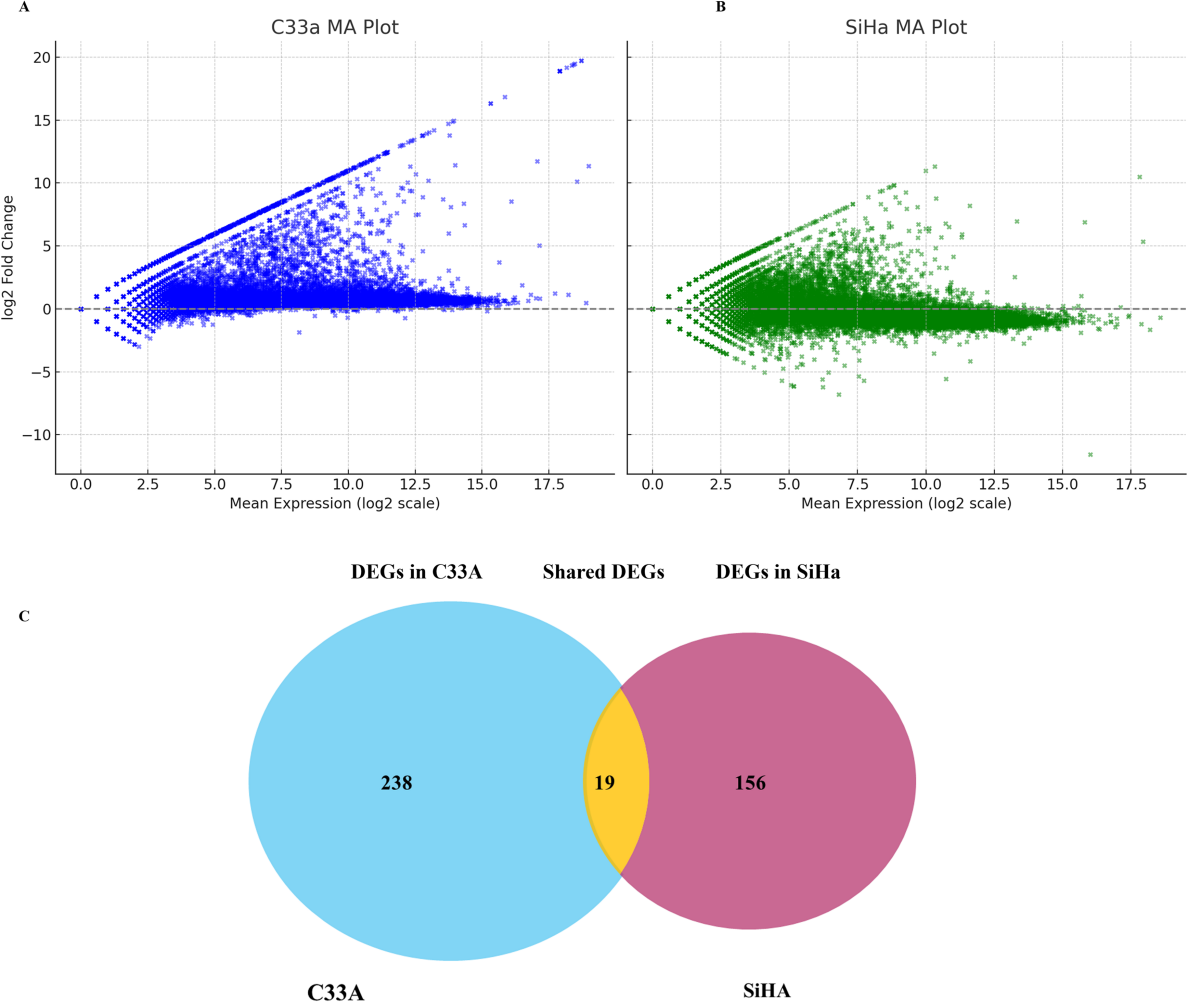
MA plots representing differential gene expression in **(A)** C33A and **(B)** SiHa CCa cell lines and **(C)** a Venn diagram following treatment. The plots display the log_2_ fold change (y-axis) against the mean expression (log_2_ scale, x-axis) for each gene. **(A)** C33A cells (blue); **(B)** SiHa cells (green). Each point represents a gene, with the horizontal dashed line indicating no change in the expression. Genes above or below this line show upregulation and downregulation, respectively. **(C)** Venn diagram of C33A and SiHa cell lines. The blue circle represents the DEGs in C33A cells, and the purple line represents the DEGs in SiHa cells. The overlapping yellow area represents the genes shared by the two cell lines.

To examine the transcriptional effect of SRPK1 inhibition, RNA sequencing was performed on C33A and SiHa cells treated with 3µM SPHINX31 or vehicle control for 48 h. Using |log_2_ FC | ≥ 1.5, 282 differentially expressed genes (DEGs) were identified in C33A (156 upregulated, 126 downregulated) and 233 in SiHa (136 upregulated and 97 downregulated). MA plots illustrated distinct transcriptional responses between the two cell lines (**Figures 1A, B**). A Venn diagram showed that C33A and SiHa cells had unique and common DEGs (**Figure 1C**).

### 3.2 Gene Ontology term enrichment analysis of DEGs in C33A

**Figure 2:**
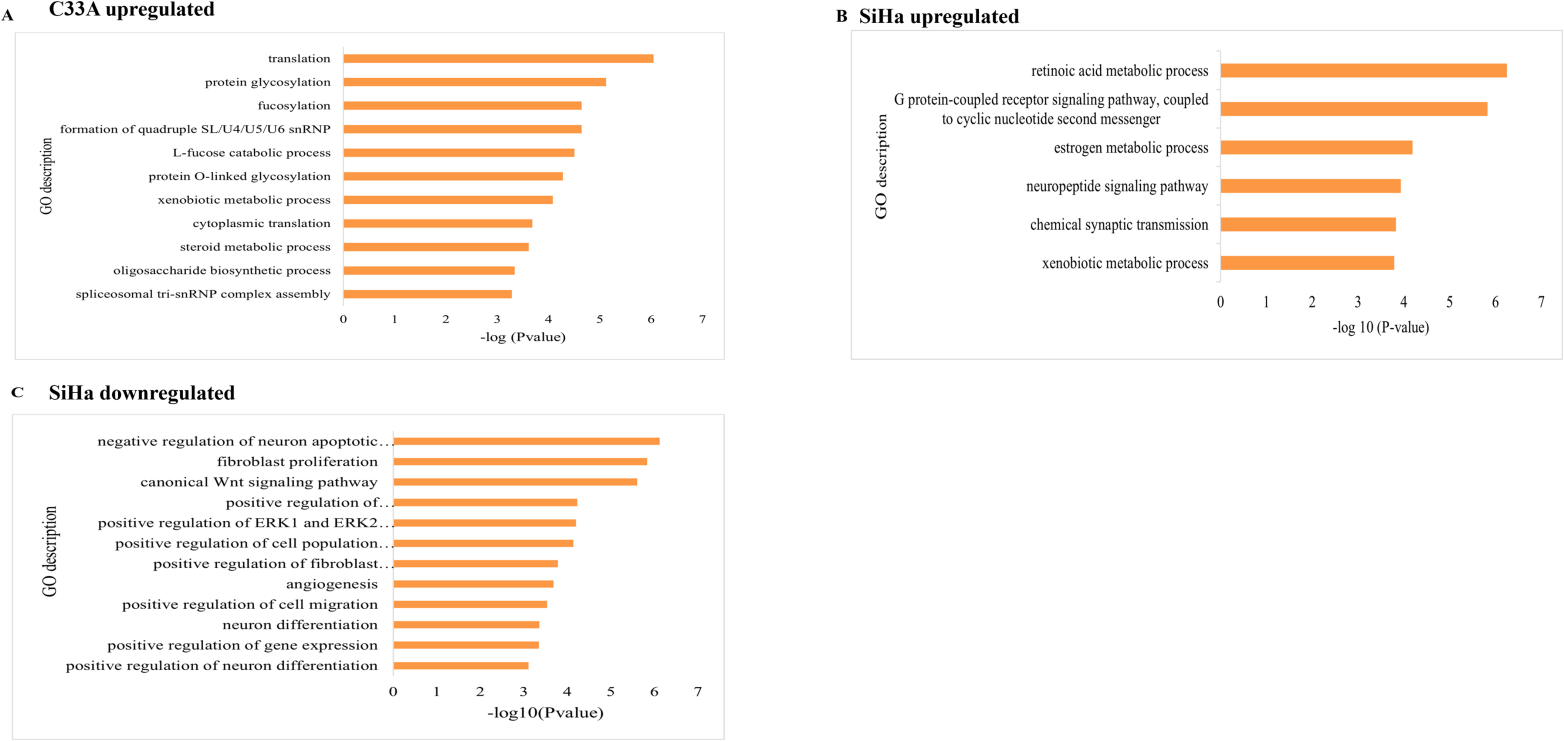
Functional enrichment of **(A)** upregulated genes in C33A, **(B)** upregulated in SiHa, and **(C)** downregulated genes in SiHa. Bar plots display the top Gene Ontology (GO) biological processes enriched among dysregulated genes in CCa and SiHa cell lines following SRPK1 inhibition. The X-axis shows enrichment strength as -log10 (P-value), while the y-axis lists GO descriptions. Substantial enrichment is indicated by longer bars arranged from most to least significant. Panel A shows enrichment among upregulated genes in C33A cells, highlighting processes involved in translation, protein glycosylation, fucosylation, formation of spliceosomal snRNP complexes (U1/U4/U5/U6), L-fucose catabolic process, protein O-linked glycosylation, cytoplasmic translation, xenobiotic and steroid metabolism, oligosaccharide biosynthetic process, and spliceosomal tri-snRNP assembly. Panel B presents enrichment among upregulated genes in SiHa cells, including G-protein-coupled receptor signaling coupled to cyclic nucleotide second messengers, neuropeptide signaling pathway, chemical synaptic transmission, retinoic acid, estrogen, and xenobiotic metabolic process. Panel C shows enrichment among downregulated genes in SiHa cells, including negative regulation of neuron apoptotic processes, fibroblast proliferation, canonical Wnt signaling, positive regulation of ERK1/ERK2 cascade, positive regulation of cell population proliferation, angiogenesis, fibroblast activation, cell migration, and neuron differentiation.

In C33A cells, GO enrichment analysis of elevated DEGs identified 13 biological process terms and 11 molecular function terms. Translation, protein glycosylation, and spliceosome assembly were the most common categories (**Figure 2A**), followed by ribosome structural components, carbonyl reductase activity, and fucosyltransferase activity (**Table 1**). Following SRPK1 suppression, this enrichment indicates an adaptive program that favors protein synthesis, RNA processing, and metabolic support. In contrast to the pathway suppression shown in SiHa (Section 3.3), C33A responses indicate a pro-translational and metabolic adaptation.

**Table 1:** Molecular function of DEGs in C33A cells.

Molecular function	Expression	P-value	Gene names
structural constituent of the ribosome	Upregulated	1.18e-10	*RNA28SN2, RNA45SN3, RNA45SN4, RNA5-8SN5, RPL17-C18orf32, RPL36A-HNRNPH2, MRPS18A, MRPS18C, RPL10L, RPL17, RPL36A, RPS10, RPS27A, RPS5*
carbonyl reductase (NADPH) activity		2.48e-05	*CBR1, CBR3, DHRS4, DHRS4L2*
fucosyltransferase activity		3.39e-05	*FUT4, FUT4, FUT5, FUT7*
oxidoreductase activity,		6.35e-05	*CYP3A7-CYP3A51P, COQ6, CYP2C19, CYP26C1, CYP3A5*
oxidoreductase activity		7.71e-05	*ALDH1A3, CBR1, COQ6, CYP2C19, CYP21A2, CYP3A5, DHRS4L2, DHRS4, DHRSX*

### 3.3 GO enrichment analysis of DEGs in SiHa cells

In SiHa cells, GO enrichment yielded a distinct pattern. Upregulated genes corresponded to five biological processes and four molecular activities, including retinoic acid metabolism, GPCR signaling, and estrogen metabolic pathways (**Figure 2B**). Downregulated genes, on the other hand, were widely dispersed throughout 11 biological processes and four molecular functions, indicating considerable reduction of canonical Wnt, PI3K/AKT, and ERK1/2 signaling, as well as fibroblast proliferation, angiogenesis, and migration (**Figure 2C**, **Table 2**). These findings indicate a coordinated downregulation of growth-factor activity, cytokine activity, and kinase signaling (**Table 2**). Thus, while C33A adapts by increasing translation and metabolic activities, SiHa suppresses carcinogenic pathways, indicating an HPV-dependent difference in SRPK1 responses.

**Table 2:** Molecular function of DEGs in SiHa cell lines.

Molecular Function	Expression	P-Value	Gene name
neurotransmitter receptor activity	Upregulated	6.35e-05	*GRIN3B, DRD4, GLRA1, HTR2B, HTR6*
transmitter-gated monoatomic ion channel activity		2.06e-04	*CHRND, CHRNE, GRIN3B, GLRA1, GRIK1*
retinoic acid binding		3.22e-04	*UGT1A9, UGT1A1, UGT1A3, CYP2W1*
iron ion binding		4.75e-04	*CYP2A7, CYP3A7, CYP3A5, CYP7A1, HAAO, CYP2W1, BBOX1*
growth factor activity	Downregulated	2.18e-05	*BMP2, BMP7, VEGFB, DGF5, NGF, PDGFRA*
signaling receptor binding		1.31e-04	*WNT16, WNT7B, BMP2, FZD8, LAMA2, LAMA4, SERPINE1*
cyclin-dependent protein serine/threonine kinase activator activity		2.74e-04	*CKS2, CCND1, CCNB1*
cytokine activity		6.88e-04	*WNT16, WNT7B, BMP2, BMP7, GDF5*

### 3.4 KEGG pathway enrichment analysis of DEGs

**Figure 3:**
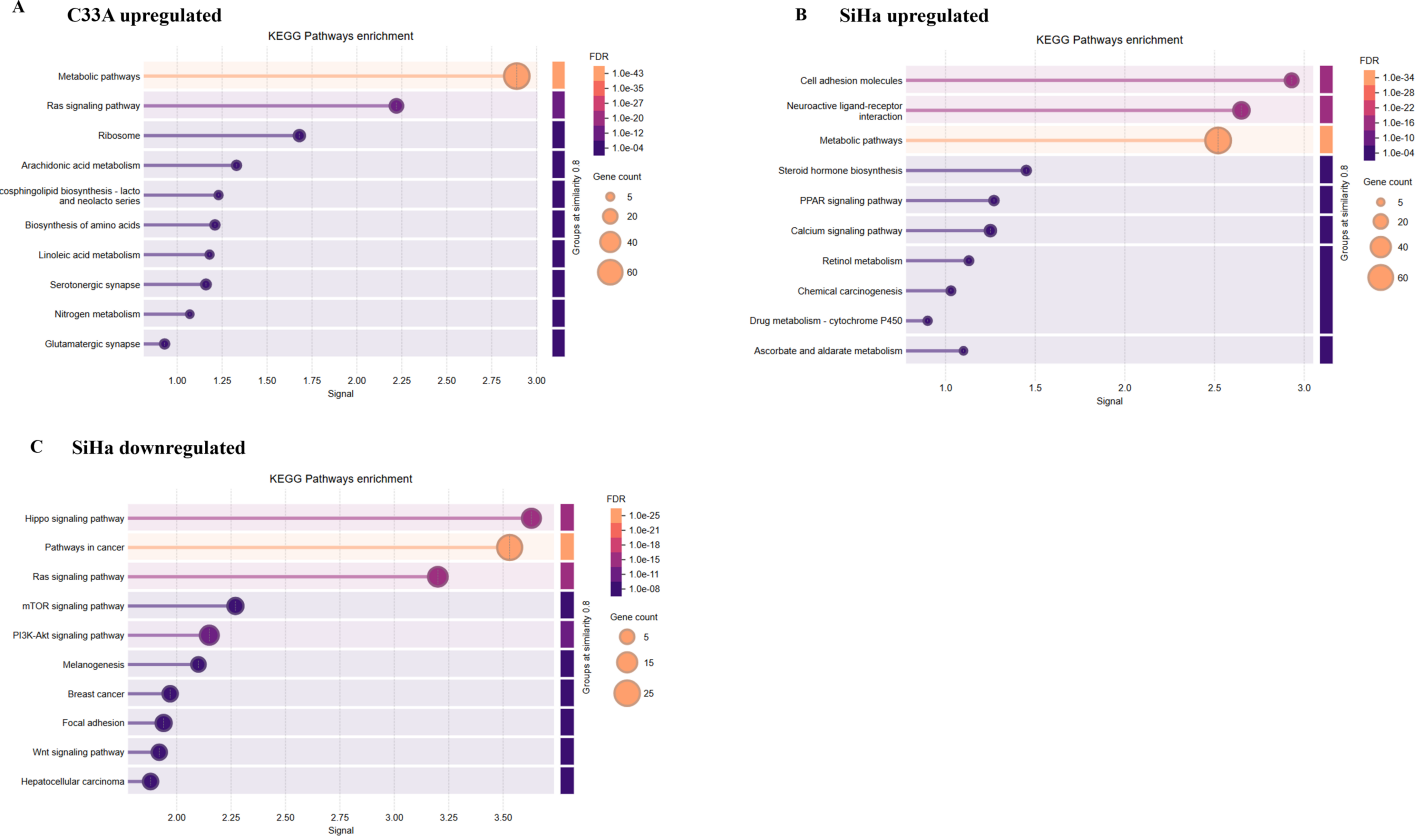
Dot plots of the top KEGG pathways enriched in the dysregulated genes in CCa and SiHa cells following SRPK1 inhibition. Dot plots display the top KEGG pathways enriched among dysregulated genes in CCa cell lines following SRPK1 inhibition. **(A)** shows pathways enriched in upregulated genes in C33A cells, including metabolic pathways, Ras signaling pathway, ribosome, arachidonic acid metabolism, phospholipid biosynthesis, biosynthesis of amino acids, linoleic acid metabolism, serotonergic synapse, nitrogen metabolism, and glutamatergic synapse. **(B)** presents pathways enriched in upregulated genes in SiHa cells, including cell adhesion molecules, neuroactive ligand–receptor interaction, metabolic pathways, steroid hormone biosynthesis, PPAR signaling pathway, calcium signaling pathway, retinol metabolism, chemical carcinogenesis, drug metabolism via cytochrome P450, and ascorbate and aldarate metabolism. **(C)** shows pathways enriched in downregulated genes in SiHa cells, including Hippo signaling pathway, pathways in cancer, Ras signaling pathway, mTOR signaling pathway, PI3K-Akt signaling pathway, melanogenesis, breast cancer, focal adhesion, Wnt signaling pathway, and hepatocellular carcinoma. Dot size represents the number of genes associated with each pathway, while the color gradient indicates statistical significance based on FDR. No significant pathways were identified among downregulated genes in C33A cells.

Following GO annotation and PPI clustering, KEGG over-representation testing was performed for C33A and SiHa DEGs to highlight pathway-level alterations. KEGG Pathway enrichment analysis of dysregulated genes in C33A revealed a notable enrichment of signaling pathways in upregulated genes. The top 10 significantly enriched pathways are shown in **Figure 3A**. Specifically, 65 of the upregulated genes were significantly enriched in metabolism-associated processes. Seventeen upregulated genes were enriched in the Ras signaling pathway. Eleven upregulated genes were enriched in the ribosomal pathway. Seven upregulated genes were significantly enriched in the arachidonic acid metabolism pathway. Five genes were significantly enriched in the glycosphingolipid biosynthesis-lacto and neolacto series pathways. Other significantly enriched pathways included the biosynthesis of amino acids, linoleic acid metabolism, serotonergic synapses, nitrogen metabolism, and glutamatergic synapses.

In contrast, the upregulated genes in SiHa cells can be classified into four categories: i) signal transduction and cell communication, including the calcium signaling pathway, PPAR signaling pathway, neuroactive ligand-receptor interaction, and cell adhesion molecule (CAMs) pathways; ii) metabolism, including metabolic pathways, ascorbate and aldarate metabolism, and retinol metabolism; iii) hormonal regulation; and iv) xenobiotic metabolism and disease association, including chemical carcinogenesis and drug metabolism (**Figure 3B**). Pathway enrichment analysis of the downregulated genes in SiHa cells identified 12 significantly enriched pathways. The Top 10 are shown in **Figure 3C**. Specifically, the downregulated genes were enriched in pathways involved in cancer, Hippo, Ras, PI3K-AKT, mTOR, focal adhesion, and Wnt signaling.

### 3.5 Protein- protein interaction network analysis and hub identification

**Figure 4:**
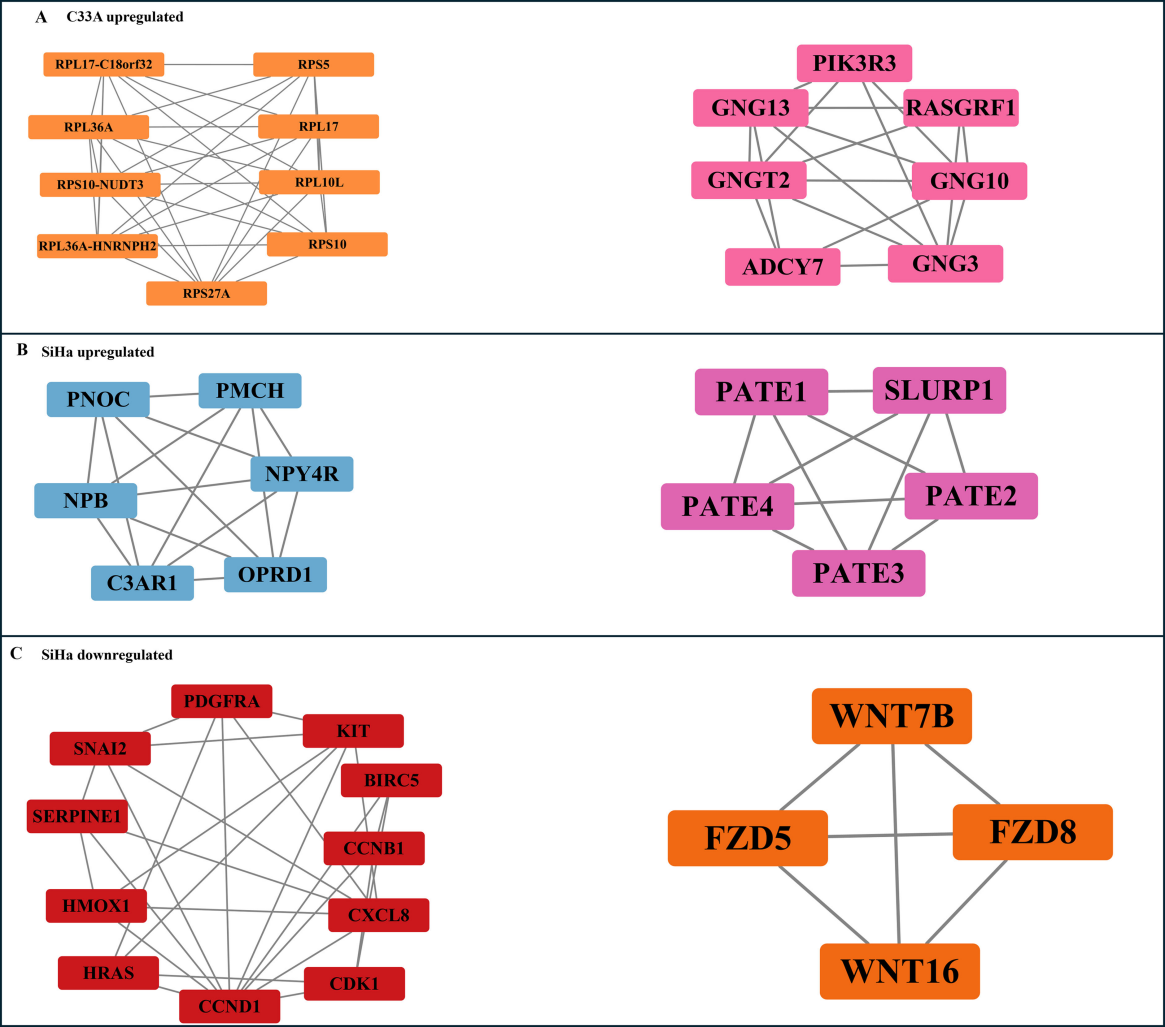
Top clusters identified using the MCODE plugin for differentially expressed genes (DEGs) in CCa cell lines following SRPK1 inhibition. **(A)** shows clusters from upregulated genes in C33A cells, including a ribosomal protein cluster (*RPL17, RPS5, RPL36A, RPS10, RPL10L, RPS27A, RPL17-C18orf32, RPL36A-HNRNPH2, RPS10-NUDT3*) and a signaling-related cluster (*PIK3R3, RASGRF1, ADCY7, GNG3, GNG10, GNG13, GNGT2*). **(B)** presents clusters from upregulated genes in SiHa cells, including a neuropeptide signaling cluster (*PNOC, PMCH, NPB, NPY4R, C3AR1, OPRD1*) and a PATE family cluster (*PATE1, PATE2, PATE3, PATE4, SLURP1*). **(C)** shows clusters from downregulated genes in SiHa cells, including one containing PDGFRA, KIT, BIRC5, *CCNB1, CDK1, CCND1, HRAS, HMOX1, CXCL8, SNAI2, and SERPINE1*, and another comprising *WNT7B, WNT16, FZD5, and FZD8*. Different colors represent distinct clusters within each DEG set.

Extending the GO findings, STRING (v12.0) PPI mapping of DEGs showed coordinated protein complexes that correspond to the enriched functionalities. MCODE analysis of the upregulated genes in C33A cells identified nine significant modules. The highest-scoring module (score =9.0; 9 nodes and 36 edges) included key hub proteins (RPL17, RPL10L, RPL36A, RPS5, RPS10, RPS27A), and ribosomal readthrough/fusion transcripts (RPL17-C18orf32, RPL36A-HNRNPH2, RPS10-NUDT3) enriched in translation (**Figure 4A**). The second hub (score =4; 7 nodes and 18 edges) included G-protein subunits (GNG13, GNGT2, GNG10, and GNG3), signaling regulator proteins (RASGRF1 and ADCY7), and a PI3K pathway protein (PIK3R3), enriched for G-protein beta-subunit binding. No significant clusters were detected among the downregulated genes in C33A cells.

In SiHa cells, the top module (score = 6; 6 nodes, and 15 edges) contained PNOC, PMCH, NPB, NPY4R, C3AR1, and OPRD1, enriched for the neuropeptide signaling pathway (**Figure 4B**). The second-highest scoring hub (score = 5; 5 nodes and 10 edges) included the PATE proteins, enriched for acetylcholine receptor regulator activity. The highest scoring downregulated hub (score =5; 11 nodes, and 27 edges) comprised cell cycle regulatory proteins (CCNB1, CCND1, and CDK1) and signaling proteins (HRAS, PDGFRA, and CXCL8, BIRC5), enriched for proliferation and migration (**Figure 4C**). The second-highest scoring hub, with a score of 4; 4 nodes, and 6 edges, included Wnt proteins such as *WNT7* and *WNT16* and Frizzled proteins (*FZD5* and *FZD8*), enriched for the canonical Wnt signaling pathway.

### 3.6 Computational docking supports SRPK1–SPHINX31 binding (PDB: 5MY8)

**Figure 5:**
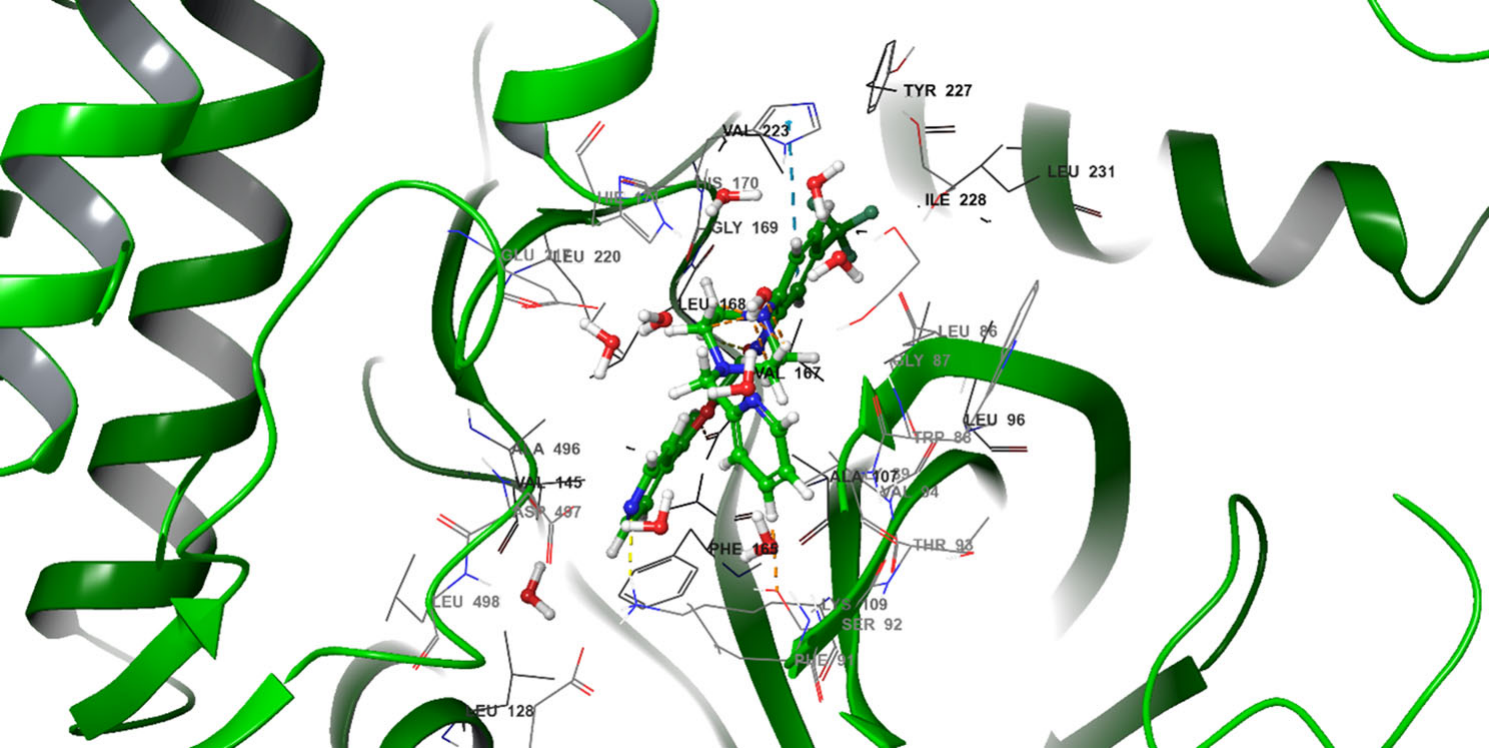
SRPK1-SPHINX31 extra-precision (XP) binding pose in the ATP site (PDB: 5MY8). A representative XP docking pose of SPHINX31 is shown bound to the canonical ATP pocket of SRPK1 from the prepared 5MY8 structure. The protein is rendered as a cartoon with a semi-transparent surface around the binding site and SPHINX31 sticks. Hydrogen-bond interactions at the kinase hinge (distances to be annotated from the model) and occupancy of the adjacent hydrophobic/back pocket are highlighted, illustrating the binding geometry characteristic of ATP-competitive kinase inhibitors. This structural view provides a mechanistic link between the small molecule and the active site that phosphorylates SR proteins, supporting the on-target engagement of SRPK1, consistent with the negative docking scores reported in **Table 3**. The pose displayed here is the same geometry used for the MM-GBSA estimation in the Results table, thereby directly connecting the visualized interactions to the computed binding free energies.

**Figure 6:**
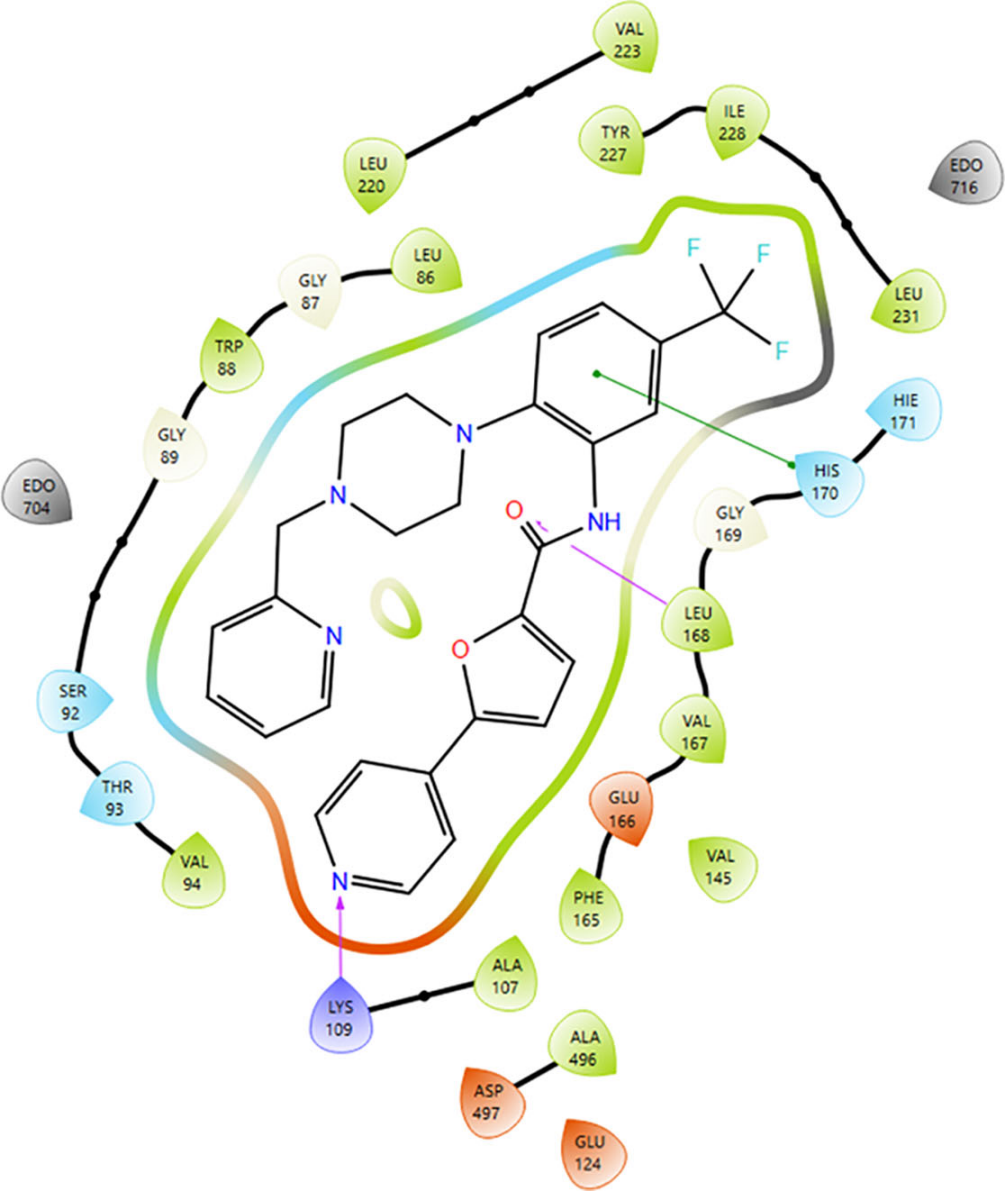
Two-dimensional ligand-protein interaction diagram for the XP pose of SPHINX31 in SRPK1 (PDB: 5MY8). This schematic translates the 3D XP pose into a clear map of the noncovalent contacts that stabilize binding in the catalytic cleft, summarizing the hydrogen-bond donors/acceptors and hydrophobic contacts between SPHINX31 and the residues lining the SRPK1 ATP pocket. In this pose, a protonated ligand nitrogen forms an electrostatic/hydrogen bond interaction with Lys109, and the ligand amide NH donates a hydrogen bond to Glu166. The ligand is enclosed by a predominantly hydrophobic wall formed by Leu86, Trp88, Val94, Val145, Phe165, Leu168, Val167, Ala107, Ala496, Leu220, Val223, Tyr227, Ile228, and Leu231, whereas Ser92, Thr93, Gly169, His170, and His171 lie at the pocket periphery. The co-crystallized re x molecules (EDO704 and EDO716) were positioned outside the main contact zone. The formation of hinge-proximal hydrogen bonds, together with complementary hydrophobic packing, is a hallmark of ATP-site engagement in kinases and provides a structural rationale for the uniformly negative docking scores and MM-GBSA ΔG bind values reported in **Table 3**. The interactions shown correspond exactly to the XP pose used for MM-GBSA, ensuring consistency between the structural visualization and energetic estimates.

To ensure that these cellular alterations were consistent with a computationally predicted interaction, computational docking of SRPK1 with SPHINX31 (PDB: 5MY8) was performed for two ligand microspecies generated at pH 7.4: one entry reported by the software without an ionization/state penalty label (“SPHINX31”) and a second entry with a state penalty of 0.0674. Standard-precision (SP) docking, extra-precision (XP) docking, and MM-GBSA rescoring were performed on the prepared 5MY8 receptor, and all metrics were reported exactly as returned by the software (**Figures 5** and **6**).

SPHINX31 exhibited a Glide XP docking score of -13.71 and -11.32 kcal/mol and an MM-GBSA binding free energy (ΔG bind) of -88.74 and -90.68 kcal/mol, indicating favorable affinity for the canonical ATP-binding pocket of SRPK1 (**Table 3**). The pose shown in **Figure 5** corresponds to this top-ranked geometry, highlighting dual hydrogen bonds to Lys109 and Glu166, as well as a hydrophobic enclosure by Leu86, Trp88, Val94, and Phe165. The 2D interaction map (**Figure 6**) summarizes these contacts. While these results predict specific on-target engagement of SRPK1, they remain computational and require experimental validation in the laboratory.

**Table 3:** Glide SP/XP docking and Prime MM-GBSA results for SPHINX31 in SRPK1 (PDB 5MY8).

Title	Ionization penalty	State penalty	Docking score	Glide emodel	Glide Gscore	XP GScore	MMGBSA dG bind	Prime energy
Glide-dock SP 5MY8 pv								
5MY8 - prepared protein								
SPHINX31	Nil	Nil	-14.708	-136.308	-14.708	Nil	Nil	Nil
SPHINX31	0.0674	0.0674	-12.639	-111.606	-12.707	Nil	Nil	Nil
Glide-dockXP 5MY8 pv								
5MY8 - prepared protein								
SPHINX31	Nil	Nil	-13.710	-125.499	-13.710	-13.710	Nil	Nil
SPHINX31	0.0674	0.0674	-11.262	-128.985	-11.329	-11.329	Nil	Nil
Prime mmgbsa 5MY8-out								
5MY8 - prepared protein								
SPHINX31	Nil	Nil	-13.710	-125.499	-13.710	-13.710	-88.740	-16005.250
SPHINX31	0.0674	0.0674	-11.262	-128.985	-11.329	-11.329	-90.680	-16030.570

The table lists the SP and XP scores (docking score, gscore, emodel, XP GScore) and MM-GBSA ΔG_bind (kcal/mol) for two Epik states: the unlabeled entry and the 0.0674 state-penalty microspecies. Both states score favorably (negative values); XP scores are more negative for the unlabeled entry, while MM-GBSA ΔG_bind is slightly more favorable for the 0.0674 state (**Figures 5**, **6**).

## 4 SRPK1 inhibition modulates alternative splicing in cervical cancer

SRPK1 inhibition was next evaluated for its effect on splicing topology. The results indicated HPV-dependent divergences, as described below by event class. AS events were systematically quantified across the two CCa cell lines, SiHa and C33A, by comparing untreated samples with SPHINX31-treated samples across all event classes (SE, RI, MXE, A3SS, and A5SS). Analysis of global splicing responses to SRPK1 inhibition revealed differences in HPV-dependent responses. SiHa cells displayed broad, fewer QC-passing events overall, with a median ΔPSI of 0.737, but a subset showed high-magnitude ΔPSI, especially at A5SS/A3SS; RI/MXE were largely unchanged. C33A showed many QC-passing events but a small effect, with most changes falling into the small-effect range (median |ΔPSI| = 0.077) and limited to selected categories. These cell line–specific patterns are described in detail below for each splicing event.

### 4.1 Exon skipping events

**Figure 7:**
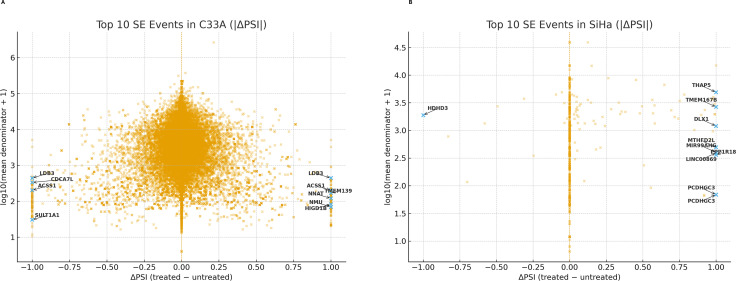
Top 10 Skipped Exon (SE) events identified in C33A and SiHa cells following SRPK1 inhibition. Scatter plots display the top 10 SE events ranked by |ΔPSI| (Percent Spliced In difference between treated and untreated conditions) in C33A **(A)** and SiHa **(B)** CCa cell lines. Each point represents an individual splicing event, plotted by ΔPSI on the x-axis and log_10_ (mean expression + 1) on the y-axis. Labeled points denote the most prominent exon skipping events in each cell line. In C33A, notable exon skipping events occur in genes such as *LDB3, ACSS1, SLIT1, HMGXB4, NNAT, and HIGD1B*. In SiHa, top exon skipping events are observed in *THAP5, TMEM167B, DLX1, MTHE2DL, PCDHGC3, and MIR99AHG*. These distributions illustrate distinct exon-skipping patterns between HPV-negative and HPV-positive CCa models in response to SRPK1 inhibition.

In SiHa cells, 1,073 SE events passed QC, with 54 events showing |ΔPSI| ≥ 0.20 and 48 exceeding |ΔPSI| ≥ 0.30. Although the overall median ΔPSI was near zero, the distribution was skewed by a subset of large-effect events, consistent with pronounced exon inclusion or skipping following SRPK1 inhibition (**Figure 7A**). In C33A cells, a much larger pool of SE events (59,234) was detected, but the majority showed minimal changes (median |ΔPSI| = 0.008). Only a minority reached the large-effect threshold, underscoring a diffuse and low-magnitude SE response compared with SiHa (**Figure 7B**). In summary, SE was widespread and low-magnitude in C33A (many QC-passing events, small median |ΔPSI|), while SiHa had fewer events but a subgroup with greater effects.

### 4.2 Alternative 3′ splice-site events

**Figure 8:**
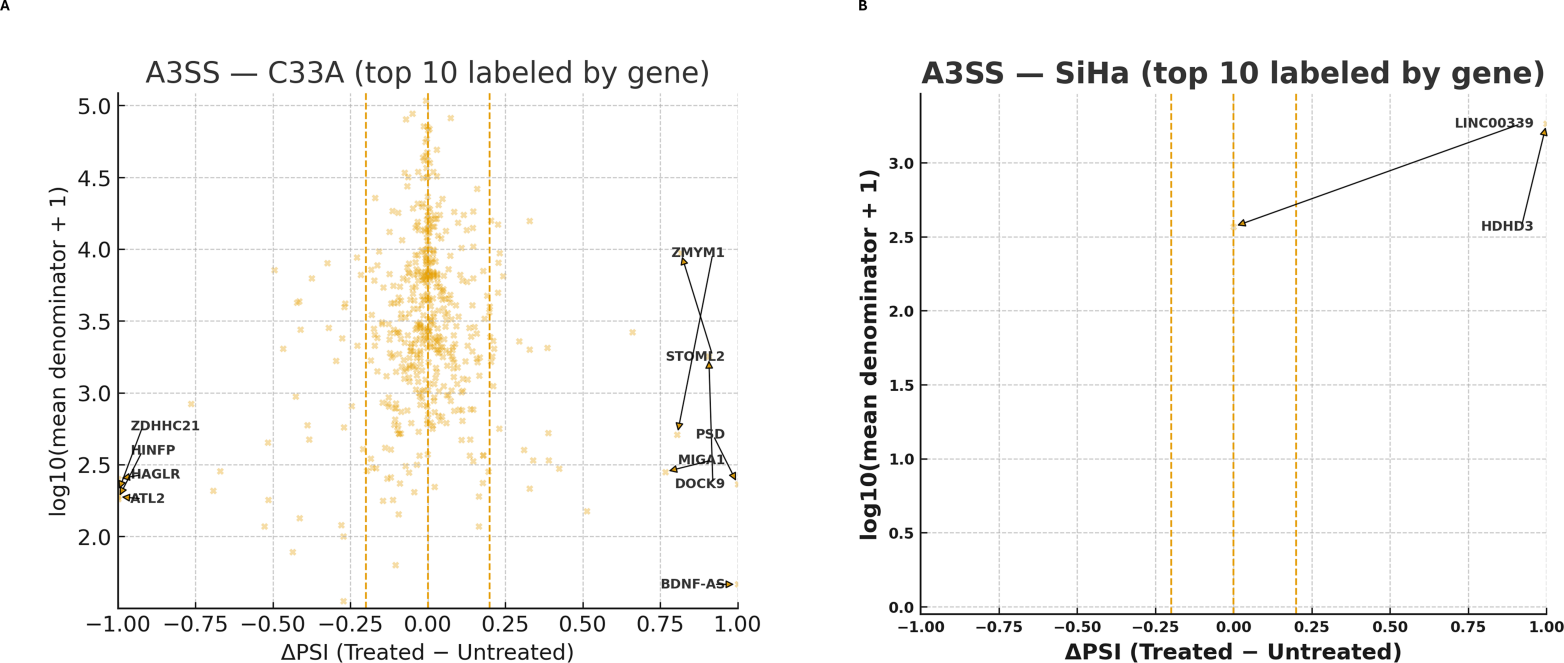
Alternative 3′ splice site (A3SS) events identified in C33A and SiHa cells following SRPK1 inhibition. Scatter plots illustrate all QC-passing A3SS events detected in **(A)** C33A and **(B)** SiHa CCa cell lines. Each orange point represents an individual splicing event plotted by ΔPSI (treated - untreated) on the x-axis and log_10_ (mean expression + 1) on the y-axis, where ΔPSI indicates the change in exon inclusion level between SRPK1-inhibited and control conditions. Vertical dashed lines mark the |ΔPSI| ≥ 0.2 threshold used to define differential A3SS events. In C33A cells, numerous genes exhibited notable shifts in splice-site usage, including *ZMYM1*, *STOML2*, *PSD*, *MIGA2*, *DOCK9*, *ATL2*, *HNRNPA1*, and *BDNF-AS*, indicating broader splice-site plasticity following SRPK1 inhibition. In contrast, SiHa cells showed relatively fewer alternative 3′ splice site alterations above the significance cutoff, with *HDHD3* and *LINC00339* emerging as the most affected transcripts. Positive ΔPSI values correspond to increased usage of distal 3′ acceptor sites, while negative ΔPSI values indicate preferential usage of proximal acceptor sites.

A3SS regulation was evident in C33A cells but not in SiHa cells. The ΔPSI values in C33A spanned both directions, with some genes showing complete splice changes (ΔPSI = ± 1). Others, including *ZMYM1*, *STOML2*, *PSD*, *MIGA2*, *DOCK9*, and *BDNF-AS*, dramatically changed toward greater distal acceptor utilization (ΔPSI = +1), whereas genes like *ZDHHC21*, *HINFP*, *HAGLR*, and *ATL2* shifted toward decreased acceptor usage (ΔPSI = -1) (**Figure 8A**). In SiHa, *HDHD3* showed a complete shift toward distal acceptor usage (ΔPSI = +1.00), whereas *LINC00339* remained unchanged (ΔPSI = 0.00). Across the top-ranked loci, the median |ΔPSI| was ~0.50, indicating selective but robust acceptor-site remodeling under SRPK1 inhibition (**Figure 8B**). Overall, A3SS remodelling was selective but robust in C33A, but negligible in SiHa, aside from isolated high-magnitude loci.

### 4.3 Alternative 5′ splice-site events

**Figure 9:**
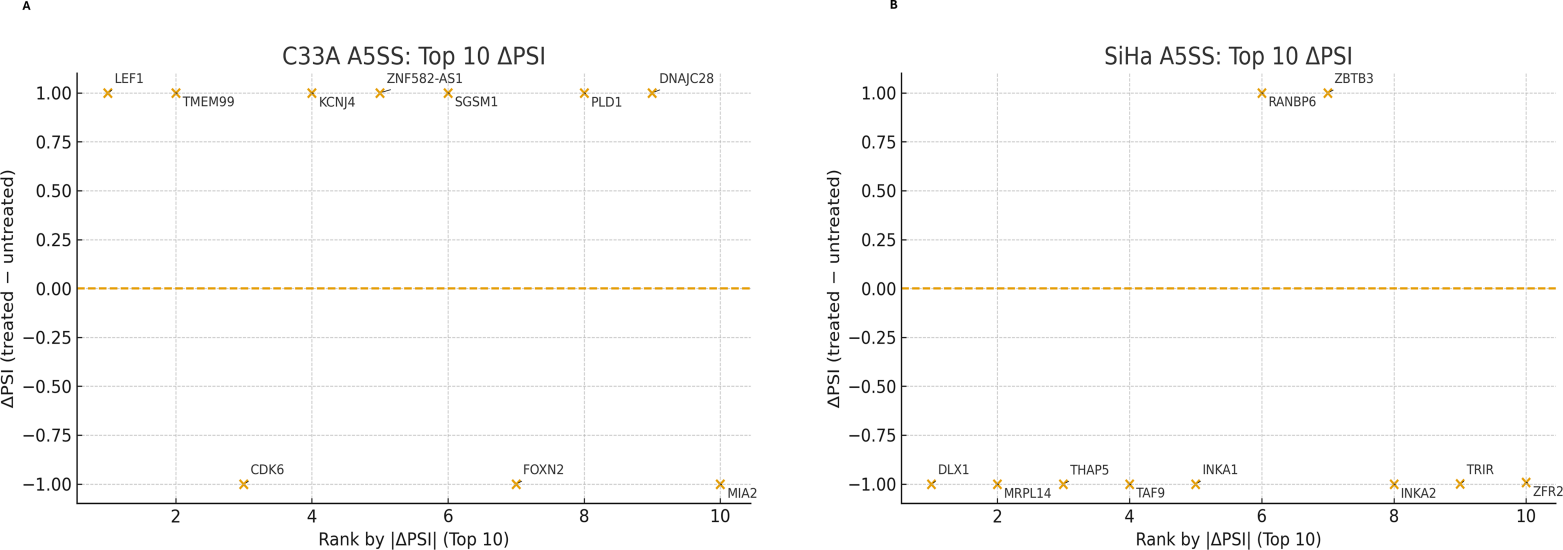
Alternative 5′ splice site (A5SS) events identified in C33A and SiHa cells following SRPK1 inhibition. Plots display the top 10 A5SS events ranked by |ΔPSI| (Percent Spliced In difference between treated and untreated conditions) in **(A)** C33A and **(B)** SiHa CCa cell lines. Each orange point represents a single A5SS event corresponding to a gene, with ΔPSI values (treated - untreated) plotted on the y-axis and ranked by absolute ΔPSI on the x-axis. The dashed horizontal line at ΔPSI = 0 indicates no change in splice-site usage. In C33A cells, the most affected genes include *LEF1*, *TMEM99*, *KCNJ4*, *ZNF582-AS1*, *SGSM1*, *PLD1*, *DNAJC28*, *CDK6*, *FOXN2*, and *MIA2*. In SiHa cells, top alternative 5′ splice site events were observed in *RANBP6*, *ZBTB3*, *DLX1*, *MRPL14*, *THAP5*, *TAF9*, *INKA1*, *INKA2*, *TRIR*, and *ZFR2*. Positive ΔPSI values denote shifts toward distal donor (5′) splice site usage, while negative values indicate a preference for proximal splice sites.

A5SS events were strongly affected in both cell lines; +ΔPSI denotes distal/long donor usage, whereas -ΔPSI denotes proximal/short donor usage. In SiHa cells, genes such as *DLX1*, *MRPL14*, and *THAP*5 exhibited complete switches away from long donor usage (ΔPSI = -1.00). In C33A cells, *LEF1* shifted from exclusive short donor usage to full-length donor inclusion (ΔPSI = +1.00), whereas *TMEM99* also stabilized the long donor variant (ΔPSI = +1.00) (**Figure 9A**). Conversely, *CDK6* lost its long donor preference (ΔPSI = -1.00). Additional high-ranking events included *KCNJ4* and *ZNF582-AS1* (ΔPSI = +1.00) (**Figure 9B**). These patterns indicate widespread 5′ splice-site remodeling under SRPK1 inhibition. Thus, A5SS regulation was prominent in both lines, but SiHa skewed toward fewer, larger-effect switches, whereas C33A demonstrated a wider occurrence with varied effect sizes.

### 4.4 Retained intron events

**Figure 10:**
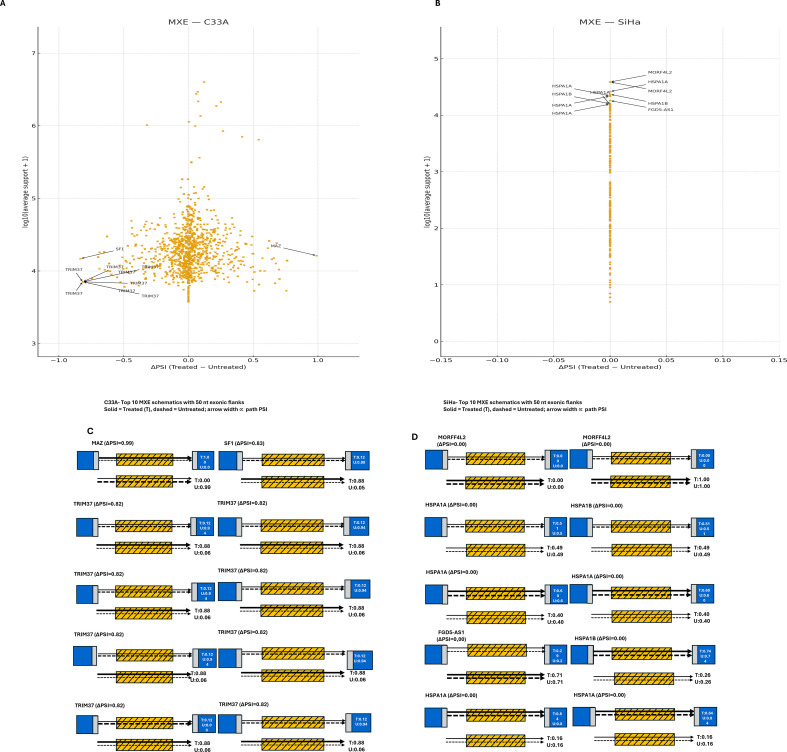
Volcano-like scatterplot of QC-passing RI events in **(A)** C33A cells and **(B)** SiHa following SRPK1 inhibition. **(A)** Volcano-like scatterplot of all QC-passing RI events in C33A cells; each orange point denotes a single intron retention event plotted by ΔPSI (Percent Spliced In difference between treated and untreated conditions) on the x-axis and log_10_(average junction support + 1) on the y-axis. Labeled examples mark the largest shifts observed, including *HLA-DRB1*, *PLIN2*, *CATSPER2*, *KLF4*, *LMO2*, *ZNF582-AS1*, *FIBCD1*, *SUGT1P-STRA6LP-CCDC180*, *FAT1*, and *CEROX1*. **(B)** Scatterplot of QC-passing RI events in SiHa cells shows a narrow distribution centered at ΔPSI ≈ 0; no event exceeded the differential-splicing threshold (|ΔPSI| ≥ 0.2), indicating minimal RI modulation in this line. **(C)** Exon-intron schematics for the top C33A intron retention events (several with ΔPSI ≈ ± 1.00), illustrating instances of complete intron retention or excision. Solid lines represent the treated condition, dashed lines the untreated condition, and arrow width depicts path PSI. **(D)** Example schematics for SiHa (*COA-AS1*, *SLC25A5-AS1*) are provided to illustrate the intron retention architecture. These events occur near ΔPSI = 0 in panel B and do not meet the |ΔPSI| ≥ 0.2 cutoff. ΔPSI > 0 indicates increased intron retention in treated vs. untreated cells, whereas ΔPSI < 0 indicates decreased retention.

RI regulation showed a clear cell line specificity. In C33A cells, 1,770 QC-passing events were detected, with several loci showing complete intron retention after SRPK1 inhibition. These included *HLA-DRB1*, *PLIN2*, *ZNF582-AS1*, *FIBCD1*, *LMO2*, *CEROX1*, and *FAM111A* (ΔPSI = +1.00). Conversely, *CATSPER2*, *KLF4*, and the multigenic locus *SUGT1P4-STRA6LP-CCDC180* lost intron retention entirely (ΔPSI = -1.00) (**Figures 10A, C**). These opposing responses demonstrate that SRPK1 enforces intron recognition fidelity in a gene-specific way. In contrast, only two RI events were detected in SiHa (*COA6-AS1* and *SLC25A5-AS1*), and both remained unchanged (ΔPSI = 0.00), indicating that RI modulation is absent in this context (**Figures 10B, D**). Overall, RI modulation was binary and common in C33A (including total retention/loss at many loci) but basically absent in SiHa.

### 4.5 Mutually exclusive exon events

**Figure 11:**
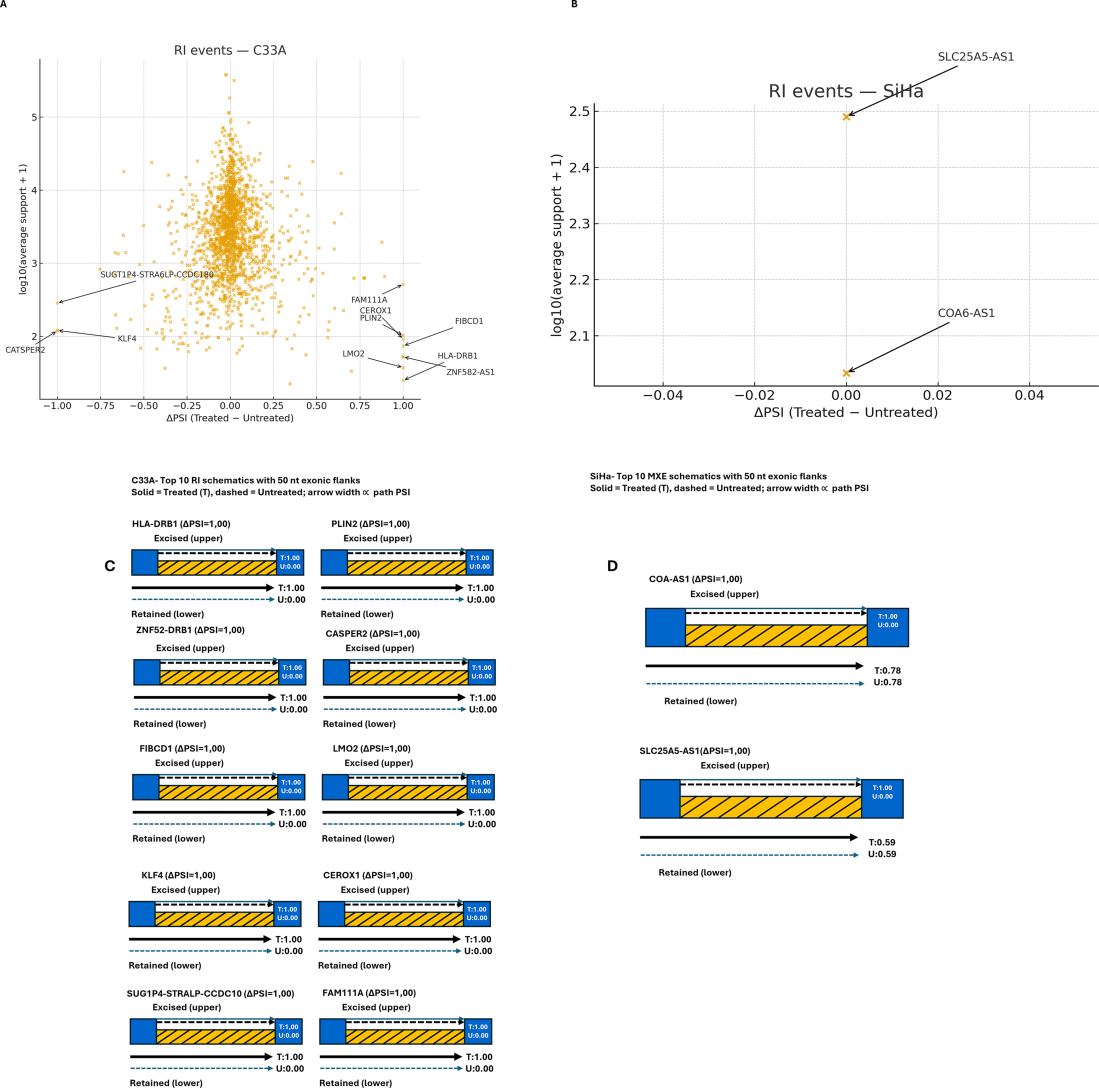
Mutually exclusive exon (MXE) events after SRPK1 inhibition. **(A)** (C33A) and **(B)** (SiHa) show volcano-style scatterplots of QC-passing mutually exclusive exon events, with each dot representing one event plotted by ΔPSI (Percent Spliced In difference between treated and untreated conditions) on the x-axis and log_10_(average junction support + 1) on the y-axis. Positive ΔPSI indicates increased inclusion of the distal mutually exclusive exon in treated cells; negative ΔPSI indicates increased inclusion of the proximal mutually exclusive exon. In C33A **(A)**, dots spread to both sides of zero, indicating bidirectional shifts in exon choice; labeled examples include *MAZ*, *SF1*, *STOML2*, and *SYNE*1. In SiHa **(B)**, dots cluster near ΔPSI ≈ 0, indicating little or no mutually exclusive exon modulation; recurrent isoforms such as *HSPA1A/HSPA1B* and *NDRFAL2* are annotated. Panels C-D provide schematics of representative mutually exclusive exon events for C33A and SiHa, respectively: blue denotes constitutive exons, orange the mutually exclusive exons, and gray the 50-nt flanks used for counting; solid arrows (DMSO-treated) and dashed arrows (untreated) trace splice paths, with arrow width proportional to path PSI. Overall, SRPK1 inhibition alters mutually exclusive exon selection in C33A but has a minimal effect in SiHa.

MXE regulation was negligible in SiHa cells but pronounced in C33A cells. Of the 4,888 QC-passing events, C33A displayed robust bidirectional changes. *MAZ* exhibited a negative shift (ΔPSI ≈ -0.989), whereas SF1 showed a positive change (ΔPSI ≈ +0.845). Additional loci included *STOML2* (ΔPSI ≈ +0.817) and *SYNE1* (ΔPSI ≈ +0.761), supporting binary exon selection under SRPK1 inhibition (**Figures 11A, C**). The events in SiHa clustered near ΔPSI = 0, showing no detectable modulation of exon usage (**Figures 11B, D**). Consequently, MXE selection was significant in C33A with bidirectional shifts, but it was minimal in SiHa.

## 5 Discussion

CCa is a prevalent female malignancy with many causes, gene variants, and a complex interplay of genetic modifications, especially HPV infections. SRPK1 contributes to oncogenic pathways across cancers and is a promising therapeutic target (31, 45–47). In this study, SRPK1 inhibition with SPHINX31 altered gene expression and AS in HPV^-^ (C33A) and HPV 16^+^ (SiHa) CCa cells, suggesting possible HPV-associated differences in SRPK1-related transcriptomic changes.

In C33A cells, SRPK1 inhibition favored translational and metabolic processes, reflected in ribosomal hubs and enrichment of RNA processing (**Figure 2A**, **Figure 3A**, and **Figure 4A**). These findings may be consistent with an adaptive cellular response focused on maintaining protein synthesis and preserving cellular homeostasis following the disruption of SR protein phosphorylation, although additional validation may be necessary to support this interpretation. This may indicate that in the absence of viral oncogenes, cells may use compensatory mechanisms to maintain protein synthesis rather than undergoing stress-mediated apoptosis. In lymphoma and myeloma, inhibiting SRPK1 induces ER stress and death through ATF4/CHOP activation and AKT suppression (48–50). The C33A response highlights a tumor-specific mechanism whereby SRPK1 inhibition maintains protein homeostasis instead of initiating death.

In contrast, SiHa cells showed a broad suppression of oncogenic signaling spanning Hippo/YAP, Wnt/β-catenin, PI3K-AKT/mTOR, Ras-ERK, focal adhesion, migration, and angiogenesis (**Figures 2C** and **3C**). The coordinated decrease in YAP/TAZ target genes (e.g., *CCND1*, *BIRC5*, *SNAI2*, *SERPINE1*) and regulators (e.g., *RASSF1*, C*SNK1E*) is consistent with attenuation of Hippo-dependent transcriptional programs that are known to be enhanced by HPV oncoproteins and growth-factor cues, enhancing proliferation, stemness, invasion, and immune evasion (51–58). PPI modules supported this suppression: one hub (*WNT7B*, *WNT16*, *FZD5*, *FZD8*) showed suppression of canonical Wnt signalling, while another hub (*CCNB1*, *CCND1*, *CDK1*) combined cell-cycle drivers (*CCNB*1, *CCND1*, *CDK1*) with signalling nodes (*HRAS*, *PDGFRA*, *CXCL8*, *BIRC5*), coherently enriched for proliferation/migration (**Figure 4C**). Given that hrHPV E6/E6*I may activate β-catenin/TCF pathways (6, 59), and that *WNT7B* facilitates HPV-mediated invasion and angiogenesis (60), the downregulation observed may be linked with the dampening of viral-amplified networks although causality cannot be established. SRPK1 inhibition was associated with a lowered expression of transcripts linked to cell cycle regulation. The inhibition of *CCND1* and *CDK1* likely compromises the G1/S and G2/M transitions (61–69). A decrease in csurvivin (BIRC5) expression may suggest changes associated with altered apoptotic responsiveness, which is often high in HPV^+^ conditions due to disruption of the p53 pathway (70).

Other tumor models show heterogeneous responses. Wang et al. found that both the upregulation and inhibition of SRPK1 might increase AKT signaling by disrupting phosphatase regulation (71), and breast cancer RNA-seq revealed approximately 187 DEGs, with enrichment for NF-κB signaling-related transcripts (*IL1A*, *IL1B*, *TRAF3*, *RELB*), indicating a function for SRPK1 in controlling inflammation and migration via transcription regulation (45). Such disparities highlight how the oncogenic effects of SRPK1 activity differ by tumour type. In this study, the suppression of HPV-linked oncogenic signaling in SiHa cells suggests a distinct viral context in which SRPK1 may promote malignant programming.

The docking analysis computationally predicted docking, consistent with SRPK1 binding by SPHINX31 (**Table 3**, **Figures 5** and **6**). The ability of SPHINX31 to occupy the ATP pocket through specific polar interactions with Lys109 and Glu166, as well as complementary burial against a leucine/valine/phenylalanine-rich surface, is consistent with ATP-site engagement by a kinase-directed inhibitor. This predicted structural interaction, which is compatible with a possible SPHINX31- SRPK1 interaction, provides a backdrop for the downstream transcriptomic and AS results presented in this study, and suggests that the cellular responses observed under SRPK1 inhibition may be compatible with predicted in silico binding in this model.

AS analysis suggested HPV-associated divergences. In SiHa (HPV16^+^), fewer but larger effect events, concentrated at A5SS and A3SS, were observed, which may be consistent with selective splice-site alteration rather than general intron definition, for example, A5SS switches included *DLX1*, *MRPL14*, and *THAP5*, raising the possibility of transcriptional regulation, mitochondrial translation, and cell-cycle/apoptosis implications (**Figures 8** and **9**) (72–75). In C33A (HPV^-^), SE changes were more widespread but mostly low-effect, whereas RI and MXE showed more binary, all-or-none patterns (complete retention or loss at multiple loci) (**Figures 7**, **10**, and **11**) and A5SS switches included *LEF1* (a Wnt/β-catenin transcription factor) and *CDK6* (a key cell cycle regulator) (**Figure 9**), raising the possibility of splicing linked effects on Wnt and cell cycle associated pathways: however the functional significance remains to be elucidated. Nonetheless, the specific functional implications of these isoforms remain to be determined. Among the A3SS loci in C33A, *STOML2* (overexpressed and prognostic in CCa) (76, 77) and *BDNF-AS* (tumor-suppressive in CCa) (78, 79) illustrate that SRPK1-associated splice-site alterations may occur in genes relevant to CCa.

Comparative evidence in other tumour models demonstrated that inhibiting SRPK1 changes the splice-site selection in VEGF165 (pro-angiogenic isoforms) (47, 80). Furthermore, inhibition with SRPKIN-1 changed pro-angiogenic VEGF-A165a to the anti-angiogenic VEGF-A165b variant (81). Beyond *VEGF*, SRPK1 inhibition alters the splicing of *MCL1*/*BIN1*/*BCL2* to produce pro-apoptotic isoforms in cholangiocarcinoma (39) and causes stress-associated cell death in lymphoma (48). SPHINX31 has been demonstrated to block the phosphorylation of SRSF1, resulting in AS and the production of the ΔEx3PD1 variant of *PD-1*, increasing T-cell antitumor responses (82). These studies highlight that, while SRPK1 inhibition can influence splice-site modification, the pathways involved vary, and in CCa, this is influenced by the HPV status.

## 6 Conclusion and future directions

This study presents an integrated exploratory analysis suggesting that SRPK1 inhibition may influence the CCa transcriptome and splicing in a manner that varies according to HPV status. The observed findings indicate that HPV16^+^ SiHa cells exhibit focused, high-magnitude splicing switches and suppression of oncogenic Hippo/YAP, Wnt, and PI3K-AKT signaling, while HPV^-^ C33A cells display broader but lower-impact splicing changes enriched in metabolic and translational pathways. These results suggest that SRPK1 inhibition may be linked to differences in viral and host networks, though the interpretation remains preliminary.

This study provides preliminary insight into possible HPV-linked variations in SRPK1-associated transcriptomic responses in CCa. While the study provided useful comparative information between HPV^-^ and HPV^+^ models, the interpretation remains exploratory due to the absence of a non-cancerous model. Nonetheless, we acknowledge that the study was limited to *in vitro* cell lines, representing only one HPV genotype, and that only a single inhibitor (SPHINX31) was tested. Rather than diminishing the impact, these considerations define the next logical steps for translation.

## 7 Future directions

Building on these insights, expansion to additional HPV genotypes, patient-derived organoids, and *in vivo* models will help validate and extend the potential relevance of SRPK1 inhibition. A comparative evaluation of different SRPK1 inhibitors, along with the functional validation of key isoform switches, will further refine biomarker-guided strategies. Incorporating non-cancerous CCa models may also assist in determining cancer-specific responses from stress responses. Ultimately, this work supports the continued research into SRPK1 and AS in HPV-driven CCa, positioning splicing modulation as a promising target.

## Data Availability

The original contributions presented in the study are included in the article/**Supplementary Material**, further inquiries can be directed to the corresponding author/s.
