# Supplementary material for: HPV-driven transcriptome and splicing rewiring under SRPK1 inhibition in cervical cancer
Source: Front Oncol. 2026 Jan 2;15:1712170. doi: 10.3389/fonc.2025.1712170 (PMC12807931; doi:10.3389/fonc.2025.1712170)
Supplement: Supplementary file 2 [file DataSheet2.docx]

**RNA-Seq Data Processing and Analysis (Commands)**

Reference genome: GRCh38 (primary assembly) with GENCODE v38 annotation (GTF).

Environment: Galaxy Europe (v22.05) for QC and trimming; Linux CLI for mapping and quantification.

Library type: rRNA-depleted total RNA; analyses performed unstranded (-s no in HTSeq).

Threads: 12 (modifiable).

**1. Quality Control and Trimming**

Tool: Trimmomatic v0.39

Command:

trimmomatic PE -threads 12 \

SAMPLE_R1.fastq.gz SAMPLE_R2.fastq.gz \

SAMPLE_R1_paired.fq.gz SAMPLE_R1_unpaired.fq.gz \

SAMPLE_R2_paired.fq.gz SAMPLE_R2_unpaired.fq.gz \

ILLUMINACLIP:TruSeq3-PE.fa:2:30:10 \

LEADING:3 TRAILING:3 SLIDINGWINDOW:4:20 MINLEN:36

**Galaxy settings:** TruSeq3-PE adapters; SLIDINGWINDOW:4:20; MINLEN:36; retain paired and unpaired reads.

**2. Alignment**

Tool: HISAT2 v2.2.1 with SAMtools v1.10+

Index build:

hisat2-build GRCh38.primary_assembly.genome.fa GRCh38_hisat2_index

Alignment:

hisat2 -p 12 --dta -x GRCh38_hisat2_index \

-1 SAMPLE_R1_paired.fq.gz -2 SAMPLE_R2_paired.fq.gz \

-S SAMPLE.sam

Post-processing:

samtools view -@ 12 -bS SAMPLE.sam | samtools sort -@ 12 -o SAMPLE.sorted.bam

samtools index SAMPLE.sorted.bam

**3. Gene-Level Counting**

Tool: HTSeq v0.13.5

**Command:**

htseq-count -f bam -r pos -s no -t exon -i gene_id \

SAMPLE.sorted.bam gencode.v38.annotation.gtf > SAMPLE.htseq_counts.txt

**4. Transcript Quantification**

Tool: StringTie v2.2.1

**Command:**

stringtie SAMPLE.sorted.bam -p 12 \

-G gencode.v38.annotation.gtf \

-e -B -A SAMPLE.gene_abund.tab \

-o SAMPLE.stringtie.gtf

Galaxy settings: Reference-guided mode (-G GENCODE v38); -e -B enabled; gene abundance table output (-A).

5. **Alternative Splicing Analysis**

Tool: SUPPA2 v2.3 (Python 3.10)

(a) Generate event definitions:

suppa.py generateEvents -i gencode.v38.annotation.gtf -o gencode.v38 -f ioe -e SE MX RI A5 A3

(b) PSI quantification:

suppa.py psiPerEvent -i gencode.v38_SE.ioe -e tpm_all.tsv -o SE_all

(c) Differential splicing (effect-size analysis):

suppa.py diffSplice -m empirical -gc \

-i gencode.v38_SE.ioe \

-e SiHa_DMSO_1.tpm.tsv SiHa_SPHINX31_1.tpm.tsv \

-o SiHa_SE -p 1000 --save-psi

Filtering criteria: |ΔPSI| ≥ 0.20; no p-value filtering (single-replicate design).

Event types analysed: SE, MXE, RI, A5SS, A3SS.

**Software Versions Summary**

FastQC v0.11.9

Trimmomatic v0.39

HISAT2 v2.2.1

SAMtools v1.10+

StringTie v2.2.1

HTSeq v0.13.5

SUPPA2 v2.3

Galaxy Europe v22.05

All analyses were performed using the same reference genome (GRCh38) and GENCODE v38 annotation to maintain consistency across HISAT2, StringTie, HTSeq, and SUPPA2. The detailed parameters above, together with version control and public reference datasets, ensure full reproducibility of the computational pipeline.
